# Who persistently consumes dietary supplements? A multifaceted analysis using South Korea’s nationally representative health and nutrition examination survey data

**DOI:** 10.3389/fnut.2023.1243647

**Published:** 2023-09-13

**Authors:** Hye-Young Kwon

**Affiliations:** Department of Public Health, Mokwon University, Daejeon, Republic of Korea

**Keywords:** dietary supplement, healthy behavior, subjective health, health promotion, Korea

## Abstract

**Objective:**

This study aimed to identify multifaceted factors affecting intake of dietary supplements among Koreans.

**Methods:**

Korean National Health and Nutrition Examination Survey (KNHANES) data from 2018 to 2020 were obtained, pertaining to functional food intake, health related behaviors and morbidities. A total of 12,031 participants representing the Korean adult population were identified into two groups: 1 year more consumer group (*N* = 4,345) vs. non-consumer group (*N* = 7,686). A logistic regression analysis was performed to analyze the predictors of dietary supplement consumptions.

**Results:**

Sociodemographic variables were associated with dietary supplement use. Participants who were female (odds ratio [OR] = 1.85; 95% CI, 1.59–2.15), older (OR = 1.06; 95% CI, 1.04–1.08), married (OR = 6.39; 95% CI, 3.44–11.85), highly educated, and high income earners consumed significantly more dietary supplements. Non-smoking (OR = 1.27; 95% CI, 1.06–1.53) and performing aerobic exercise (OR = 1.28; 95% CI, 1.13–1.46) predict dietary supplement consumption. Self-rated health status and health-related quality of life increased the likelihood of dietary supplement intake by 1.86 (OR = 1.86; 95% CI, 1.04–3.32) and 7.77 times (OR = 7.77; 95% CI, 1.66–33.40), respectively. The number of chronic diseases, cancer, or stroke was not significantly associated with intake. Those with obesity tended to less consume dietary supplements (OR = 0.85; 95% CI, 0.74–0.97). Hypertension (OR = 3.14; 95% CI, 1.36–7.21), osteoporosis (OR = 1.47; 95% CI, 1.11–1.95), and asthma (OR = 0.44; 95% CI, 0.27–0.73) were significantly associated with the intake.

**Conclusion:**

Considering that healthy behaviors and subjective health led to the consumption of dietary supplements, whereas current disease or catastrophic experience did not, the intake of dietary supplements should be included in health promotion in Korea.

## Introduction

The coronavirus disease 2019 (COVID-19) pandemic has led to an increased focus on personal health and well-being. As information and resources on health management overflowed via the Internet and social media, people tended to actively manage their health and reduce health risks through health-promoting practices, such as regular exercise, healthy diet, or supplement consumption including functional foods and dietary supplements.

Demand for dietary supplements has been boosted by the high-risk viral disease (1), resulting in a substantial growth of the global market of dietary supplements from USD 119.7 billion in 2019 to USD 146.8 billion in 2022 (a compound annual growth rate 7.0%), and it is expected to reach approximated USD 218.4 billion by 2027 (2).

Korea is a one of the countries facing significant challenges due to aging (3). The life expectancy of the Korean population is 83.6 years, and the proportion of the elderly population reached 17.5% in 2022 and is expected to increase to 40.1% in 2050 (4). Moreover, as of 2021, the total fertility rate ranked the lowest, 0.81, among Organisation for Economic Co-operation and Development countries (3). Aging populations with longer life expectancies have become more conscious about managing their health and well-being. In particular, the increased interest in immune-boosting foods or supplements owing to the COVID-19 pandemic has led to the consumption of dietary supplements. According to the Ministry of Food and Drug Safety, the Korean functional food (dietary supplement) market has reached approximately KRW 4.4 trillion in 2020, an increase of 13.8% from the previous year (5).

In general, functional foods are defined as foods that are either enriched or fortified to restore pre-processed nutrient levels (enriched flour), to improve the nutritional quality of nutrient-deficient foods (calcium in orange juice), or to address public health issues (vitamin D in milk, iodized table salt), while dietary supplements, unlike functional foods, are defined as concentrated forms of food-derived nutrients that are designed to be taken in addition to daily food consumption for additional nutrients or perceived health benefits (6). According to the Federal Food, Drug, and Cosmetic Act in the US, a dietary supplement is defined as a product taken by mouth that contains a “dietary ingredient” intended to supplement or enhance the diet. The “dietary ingredients” in these products may include: vitamins, minerals, herbs or other botanicals, amino acids, and substances such as enzymes, organ tissues, glandulars, and metabolites. Dietary supplements can also be extracts or concentrates, and may be found in many forms such as tablets, capsules, soft gels, gel caps, liquids, or powders (7, 8). However, these distinctive definition is not applicable in Korea.

According to the Korean Functional Food Act, “functional food” refers to foods or food-driven products manufactured or processed with useful functional raw materials or ingredients for modifying the physiological functions, maintaining homeostasis, or improving specific physiological parameters. The term “functionality” means utilizing nutrients to affect the physiological functions of the human body or providing useful effects for hygiene purposes, including psychological benefits (9). However, “dietary supplement” has no official definition by health authorities in Korea. It was defined only for the Korean Health and Nutrition Examination Survey (KNHANES) as “tablets, capsules, powders, granules, liquids, and pills containing vitamins, minerals, and functional ingredients that include medicines, functional foods and products containing vitamins, minerals and functional ingredients that are not yet approved as functional foods. Dietary supplements do not include fortified foods, infant formulas, special nutritional foods such as enteral nutrition, herbal medicines, and products prepared by food processors” (10). Thus, these two terms, dietary supplements and functional foods, are not perceived differently in Korea. Only functional foods, which are considered dietary supplements, are formally regulated by the MFDS.

Several studies have investigated the factors contributing to functional food or dietary supplement intake, mainly focusing on gender, age, socioeconomic factors, and health status (11–23). Although the results are not generalizable (11, 12), and are even somewhat contradictory (13, 22, 23), women, older adults, and those with higher levels of education and income were more likely to consume functional foods or dietary supplements (14–18). Health-conscious people committed to managing their health, such as through physical activity or healthy eating, were more likely to consume functional foods or dietary supplements (16, 18–22). However, findings are inconsistent, with a study showing that people in good health consumed more functional foods or supplements (11), whereas other studies have shown that those with lower subjective health (12) or those with obesity, chronic illnesses, and disabilities consumed more (14, 18). Previous research confirmed that the consumption of functional foods was influenced, whether consistent or inconsistent, not only by sociodemographic factors but also by psychosocial and cultural factors, which was again identified by Mullie et al. (19) and Frewer et al. (20), who reported a cross-cultural difference in consumers’ attitudes toward functional foods that led to their consumption. Using nationally representative NHANES data, researchers revealed that U.S. adults used dietary supplements to improve or maintain overall health (18, 21), and supplement users were more likely than non-users to report very good or excellent health, a balanced diet, regular doctor visits, good sleep, moderate alcohol use, abstinence from smoking, and more frequent exercise (18, 21).

However, there is a dearth of research on dietary supplement consumption in Korea. Choi et al. (22) examined the significant factors contributing to the use of functional foods using data from the Korean Health Panel in 2008. Choi and his colleagues found that Korean adults with a higher educational level, more stable economic status, and chronic diseases were more likely to use dietary supplements. Unlike in other studies, females tended to adopt less dietary supplements. Health-related factors such as having disability and chronic diseases, having being admission, and physician visits were significantly associated with dietary supplement consumption (22). Park et al. revealed that educational level critically influenced dietary supplement intake among menopausal women (23).

Therefore, this study sought to elucidate the determinants of dietary supplement intake among Koreans, which could have implications for public health interventions, policymaking, and future research in the field of dietary supplements. To this end, the nationwide representative KNHANES data was used to analyze multifaceted factors, including sociodemographic factors and health behaviors that have been mainly observed in previous studies, as well as health status and morbidities that influence dietary supplement intake, to ensure the generalizability of their findings to the Korean population as a whole.

## Methods

### Data source

The KNHANES is a large-scale nationwide survey conducted annually by the Ministry of Health and Welfare and Korea Disease Control and Prevention Agency. Its purpose is to generate statistical data on the general health and nutritional status of South Koreans and to identify population groups that should be prioritized when considering health policies. A stratified multistage probability sampling design that considers location and residence type was used in the KNHANES to establish nationwide representativeness (24). This study was based on 3 years data obtained from the KNHANES 2018–2020 (24, 25), on dietary supplements and duration of supplement use.

**Figure 1:**
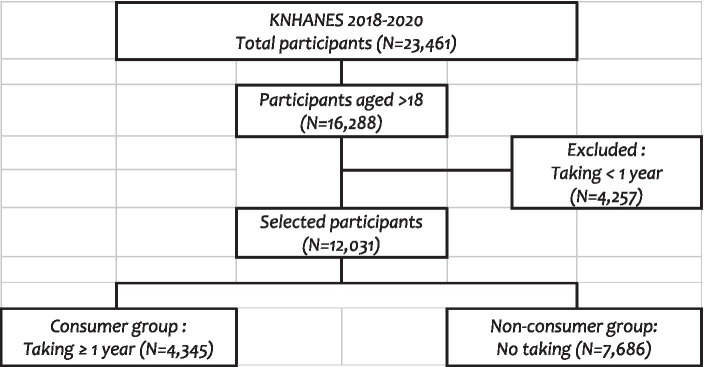
Selection process of the study population.

From the 16,288 KNHANES participants in the cross-sectional surveys conducted over the 3 years period and pooled for this analysis, 12,031 were selected. A group of 4,345 participants who reported dietary supplement consumption for at least 1 year (consumer group ≥1 year) was compared with 7,686 participants who never consumed dietary supplements (non-consumer group) (Figure 1).

All data were downloaded from the official website of the KNHANES,^1^ which is open to the public after completing a designated registration process for access.

### Sociodemographic variables

Demographic and socioeconomic variables were considered as confounders. Age, sex, marital status (e.g., married or single), and number of family members were included in the analysis. In addition, education level (e.g., primary school, middle school, high school, and bachelor’s degree or higher), income level (e.g., low, middle-low, middle-high, and high), and employment status were included in the analysis.

### Variables related to health behaviors

We included the following variables related to health behavior that were surveyed in the KNHANES: Current smoking was defined as having smoked at least 5 packs of tobacco (100 cigarettes) in their lifetime and still smoking. Monthly drinking was defined as drinking at least once per month in the past year. In addition, walking for at least 30 min once daily for at least 5 days in the past week, performing aerobic physical activities, and using ambulatory services within the last 2 weeks (26).

### Health status

Variables indicating current health status were selected for the analysis. Self-rated health status on a 5-point Likert scale shows the participants’ subjective health status. The EuroQol 5-Dimensions (EQ-5D) index score, which represents health-related quality of life (HRQoL), ranging from 0 (death) to 1 (perfect health), was calculated using the South Korea-specific tariff based on the time-tradeoff method (27, 28). Body mass index (BMI) (kg/m^2^) was calculated based on the height and weight of the participants.

### Predisposing diseases or comorbidities

The chronic diseases that the participants indicated they currently had including hypertension, diabetes, dyslipidemia, osteoporosis, arthritis (either osteoarthritis or rheumatoid arthritis), asthma, thyroid disorders, renal disorders, atopic diseases, hepatitis B or C, cirrhosis, depression, sinusitis, and otitis, were considered. The number of chronic diseases currently affecting the participants’ health was calculated using the above-mentioned diseases. In addition, previously diagnosed cancers, stroke, myocardial infarction (MI), or angina that may affect the current health status were also identified. Lastly, obesity (BMI ≥25) was also considered for analysis.

### Statistical analysis

To identify factors contributing to the quality of life, multivariate logistic regression was performed using the PROC SURVEYFREQ, PROC SURVEYMEANS and SURVEYLOGISTICS (26, 29). In the multivariate logistic regression model, the dependent variable was the intake of dietary supplements for at least 1 year or not at all (consumer group versus non-consumer group). The variables above mentioned were considered as independent variables. The statistical significance level was set to 5%. SAS version 9.4 (SAS Institute Inc., Cary, NC, United States) was used for statistical analysis.

### Ethical statement

The institutional review board (IRB) of the Korea Centers for Disease Control and Prevention reviewed and approved the KNHANES survey annually (approval numbers: 2018-01-03-P-A, 2018-01-03-C-A, and 2018-01-03-2C-A) (25). This retrospective study was approved by the ethics committee of Mokwon University (IRB no. Mokwon 2023-002).

## Results

### Basic characteristics

Table 1 shows the basic characteristics of the study population (intake group vs. no taking). The consumer group included significantly more women (57.62% vs. 44.58%) (*p* < 0.0001) and those of older age than the non-consumer group [51.81(SE0.34) vs. 46.46(SE0.33) years; *p* < 0.0001].

**Table 1:** Basic characteristics of the study population.

Variables		Total (%)		Consumer group		Non-consumer group		*p*-value
				*N*	(%)	*N*	(%)	
Sex, %	Male	5,334	(43.14)	1,578	(42.38)	3,756	(55.42)	<0.0001
	Female	6,697	(56.86)	2,767	(57.62)	3,930	(44.58)	
Age (yrs)	Mean (SE)	48.32	(0.27)	51.81	(0.34)	46.46	(0.33)	<0.0001
Age group (yrs), %								
	~29	1,351	(8.29)	182	(6.44)	1,169	(21.81)	<0.0001
	30 ~ 39	1,490	(9.15)	498	(14.26)	992	(15.67)	
	40 ~ 49	2,071	(12.71)	790	(20.91)	1,281	(17.91)	
	50 ~ 59	2,159	(13.26)	965	(24.71)	1,194	(17.17)	
	60 ~ 69	2,288	(14.05)	1,004	(19.54)	1,284	(13.35)	
	70+	2,672	(16.40)	906	(14.14)	1,766	(14.09)	
Education Level								
	Elementary school	2,146	(13.18)	636	(11.55)	1,510	(15.09)	<0.0001
	Middle school	1,061	(6.51)	399	(8.04)	662	(7.69)	
	High school	3,593	(22.06)	1,254	(32.48)	2,339	(39.13)	
	Bachelor or higher	3,955	(24.28)	1,646	(47.93)	2,309	(38.09)	
Size of family, %								
	1	1,578	(9.69)	529	(9.87)	1,049	(10.96)	<0.0001
	2	3,833	(23.53)	1,556	(30.11)	2,277	(24.37)	
	3	2,982	(18.31)	1,086	(27.59)	1,896	(27.55)	
	4	2,672	(16.40)	881	(24.91)	1,791	(27.60)	
	5	749	(4.60)	220	(5.69)	529	(7.81)	
	6 +	217	(1.33)	73	(1.82)	144	(1.70)	
Marital status, %								
	Married	9,918	(60.89)	3,907	(86.35)	6,011	(70.04)	<0.0001
	Single	2,113	(12.97)	438	(13.65)	1,675	(29.96)	
Income, %								
	Low	3,054	(18.75)	833	(18.70)	2,221	(28.71)	<0.0001
	Middle-low	2,983	(18.31)	1,006	(23.37)	1,977	(25.75)	
	Middle-high	2,994	(18.38)	1,118	(25.98)	1,876	(24.72)	
	High	2,959	(18.17)	1,378	(31.96)	1,581	(20.82)	
Employment status, %								
	Employed	6,343	(38.94)	2,331	(63.80)	4,012	(62.24)	<0.0001
	Not-employed	4,397	(27.00)	1,599	(36.20)	2,798	(37.76)	

The consumer group had higher education (*p* < 0.0001), income (*p* < 0.0001), and employment rates (*p* < 0.0001) than the non-consumer group. In the consumer group, 86.35% were married, whereas in the non-consumer group, 70.04% were married (*p* < 0.0001). The non-consumer group had more family members than the consumer group (*p* < 0.0001).

### Consumption of dietary supplements

**Figure 2:**
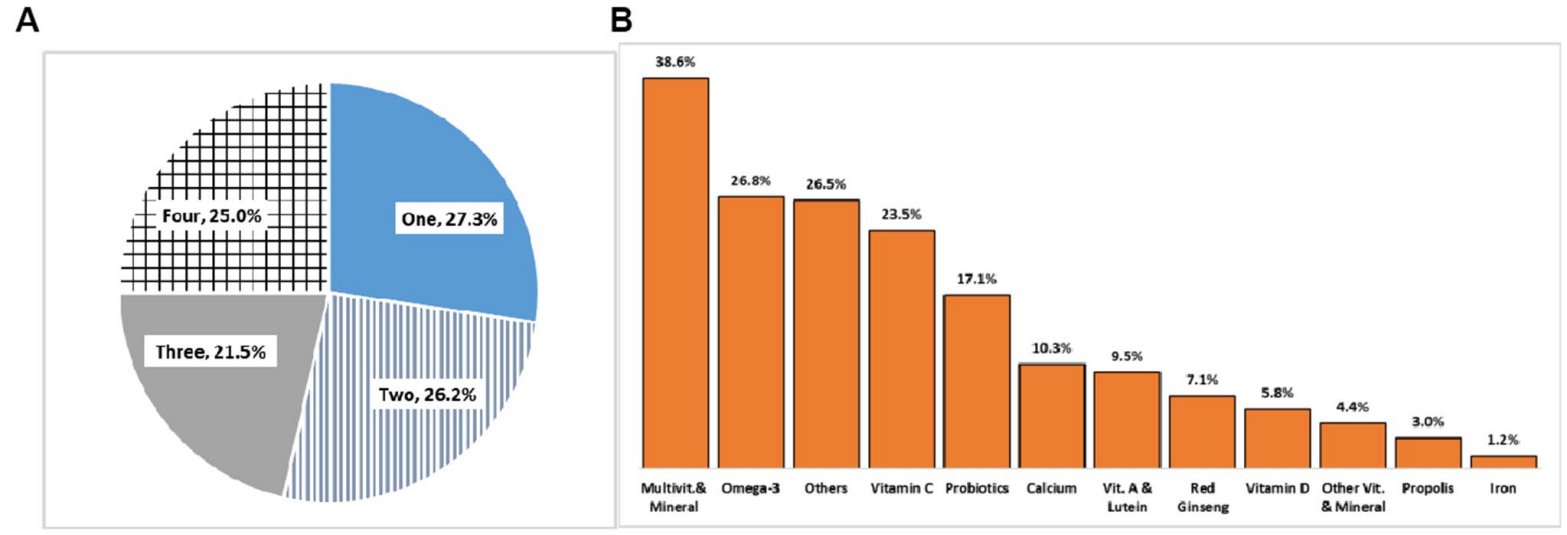
Number of different types of dietary supplement intake and percentage of respondents **(A)** and the frequency **(B)**.

The consumer group adopted an average of 2.45(SE 0.02) types of dietary supplements, which might have been underestimated because the KNHANES questionnaires allowed the respondents to enter a maximum of four products. Of these, 27.3% took one type, 26.2% took two types, 21.5% took three types, and 25% took four types (Figure 2A). Therefore, most participants in the consumer group tended to take several ingredients simultaneously. The most frequently consumed were multivitamins and minerals (38.6%), followed by omega-3 (26.8%), others (26.5%), and vitamin C (23.5%) (Figure 2B).

### Health behaviors

**Figure 3:**
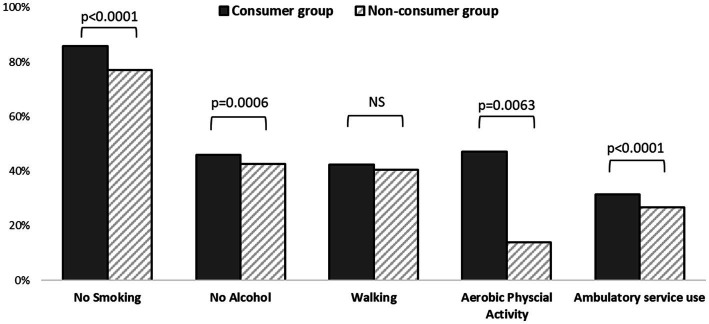
Comparison of health behaviors between the consumers and non-consumers (unadjusted).

As shown in Figure 3, all variables related to health behaviors differed significantly between the two groups, except for walking practice. Those who consumed dietary supplements tended to manage their health more effectively. In other words, those who took care of their health by refraining from smoking or drinking, practicing physical exercise, or using outpatient services tended to consume the supplements.

### Health status

Table 2 shows that subjective health status such as self-rated health or HRQoL was higher in the consumer group than in the non-consumer group (*p* < 0.0001). Meanwhile, the non-user group had higher BMI (24.17, SE 0.06) than the user group (23.8 SE 0.06) (*p* < 0.0001).

**Table 2:** Health status between the two groups (unadjusted).

Variables	Total (%)		Consumer group		Non-consumer group		*p*-value
			*N*	(%)	*N*	(%)	
Self-rated health, Mean (SE)	3.16	(0.01)	3.19	(0.02)	3.16	(0.01)	<0.0001
EQ-5D index, Mean (SE)	0.95	(0.00)	0.96	(0.00)	0.95	(0.00)	<0.0001
BMI, Mean (SE)	24.04	(0.04)	23.80	(0.06)	24.17	(0.06)	<0.0001

### Predisposing diseases or comorbidities

The consumer group had an average of 0.94 (SE 0.02) (min 0-max 8) of chronic diseases, including hypertension, diabetes, dyslipidemia, osteoporosis, arthritis, asthma, thyroidal disorders, renal diseases, atopic diseases, hepatitis B, cirrhosis, depression, sinusitis, and otitis that currently affect their health, whereas the non-consumer group has an average of 0.80 (SE 0.02) (min 0-max 10; *p* < 0.0001) (Table 3).

**Table 3:** Prevalence of chronic diseases or diagnosis of predispoing diseases between the two groups (unadjusted).

Variables		Total (%)		Consumer group		Non-consumer group		*p*-value
				*N*	(%)	*N*	(%)	
No. of chronic diseases, Mean (SE)		0.85	(0.01)	0.94	(0.02)	0.81	(0.02)	<0.0001
Chronic diseases, Yes (%)		6,267	(52.09)	2,395	(49.84)	3,872	(43.64)	<0.0001
	Hypertension	2,920	(24.27)	1,062	(21.41)	1,858	(19.32)	0.0171
	Diabetes	1,233	(10.25)	431	(9.06)	802	(8.36)	NS
	Dyslipidemia	2,011	(16.72)	866	(18.36)	1,145	(12.63)	<0.0001
	Osteoporosis	789	(6.56)	341	(6.37)	448	(4.21)	<0.0001
	Arthritis	1,366	(11.35)	547	(10.66)	819	(8.53)	0.0003
	Asthma	207	(1.72)	61	(1.07)	146	(1.82)	0.0030
	Thyroidal disorder	263	(2.19)	125	(2.71)	138	(1.76)	0.0031
	Renal disorder	101	(0.84)	46	(0.97)	55	(0.69)	NS
	Atopic diseases	1,936	(16.09)	702	(17.10)	1,234	(17.31)	NS
	Hepatitis B	123	(1.02)	49	(1.28)	74	(0.96)	NS
	Hepatitis C	28	(0.23)	11	(0.24)	17	(0.18)	NS
	Cirrhosis	43	(0.36)	10	(0.29)	33	(0.41)	NS
	Depression	326	(2.71)	125	(2.82)	201	(2.54)	NS
	Sinusitis	222	(1.85)	78	(1.86)	144	(2.34)	NS
	Otitis	105	(0.87)	39	(0.89)	66	(0.94)	NS
Stroke		281	(2.34)	82	(1.63)	199	(2.14)	NS
Myocardial infarction or angina		373	(3.10)	123	(2.53)	250	(2.57)	NS
Cancer		658	(5.47)	291	(9.14)	367	(6.27)	<0.0001
Tuberculosis		406	(3.37)	171	(3.72)	235	(3.04)	NS
Obesity		4,031	(33.51)	1,351	(32.63)	2,680	(37.74)	<0.0001

*Bold values mean statistically significant.

Accordingly, 49.8 and 43.6% of the consumer and non-consumer groups, respectively, reported to have at least one chronic disease (*p* < 0.0001). More specifically, hypertension (21.41% vs. 19.32%, *p* = 0.0171), dyslipidemia (18.36% vs. 12.63%, *p* < 0.0001), osteoporosis (6.37% vs. 4.21%, *p* < 0.0001), arthritis (10.66% vs. 8.53%, *p* = 0.0003), and thyroid disease (2.71% vs. 1.76%, *p* = 0.0031) were significantly more prevalent in the consumer group than in the non-consumer group. In contrast, asthma was more prevalent in the non-consumer group (1.07% vs. 1.82%, *p* = 0.0030). Stroke occurred more frequently in the non-user group than in the consumer group (1.63% vs. 2.14%, *p* = 0.0689), whereas tuberculosis was more frequent in the consumer group than in the non-user group (3.72% vs. 3.04%, *p* = 0.0882). However, these adverse events were marginally significant. Cancer was diagnosed more frequently in the user group than in the non-user group (9.14% vs. 6.27%, *p* < 0.0001), whereas obesity was more frequent in non-users than in users (37.74% vs. 32.63%, *p* < 0.0001).

### Factors contributing to the intake of dietary supplements

Multivariate logistic analyses revealed that in model I, where chronic diseases were presented as the number of chronic diseases currently affecting the study population, female participants were 1.85 times more likely than male participants (OR = 1.85; 95% CI, 1.59–2.15) to consume dietary supplements (Table 4). Aging by 1 year increased the likelihood of dietary supplement intake by 1.06 times (OR = 1.06; 95% CI, 1.04–1.08). Those who were married were 6.39 times more likely to consume dietary supplements than those who were unmarried (OR = 6.39; 95% CI, 3.44–11.85). Furthermore, those with higher educational level and income level had higher likelihood of dietary supplement intake. In contrast, the size of family lowered the likelihood of the intake (OR = 0.89; 95% CI, 0.83–0.94). Employment status was not a statistically significant factor.

**Table 4:** Results of multivariate logistic analysis.

Variables			Model I			Model II		
			OR	95% CI		OR	95% CI	
	Intercept		0.00	0.00	0.01	0.00	0.00	0.01
Socio-demographics	Sex (Ref = Male)	Female	**1.85**	**1.59**	**2.15**	**1.81**	**1.55**	**2.11**
	Age		**1.06**	**1.04**	**1.08**	**1.06**	**1.04**	**1.07**
	Education Level	Middle school	**1.69**	**1.34**	**2.13**	**1.64**	**1.30**	**2.06**
	(Ref = Elementary)	High school	**2.58**	**2.07**	**3.22**	**2.53**	**2.04**	**3.13**
		BA or higher	**3.31**	**2.59**	**4.24**	**3.27**	**2.57**	**4.15**
	Size of family		**0.89**	**0.83**	**0.94**	**0.88**	**0.83**	**0.94**
	Marital status(Ref = Single)	Married	**6.38**	**3.44**	**11.84**	**5.33**	**2.84**	**10.00**
	Income (Ref = Low)	Middle-low	**1.46**	**1.19**	**1.79**	**1.46**	**1.19**	**1.79**
		Middle-high	**1.55**	**1.25**	**1.91**	**1.55**	**1.25**	**1.92**
		High	**1.98**	**1.60**	**2.44**	**1.97**	**1.60**	**2.44**
	Employment	Employed	1.12	0.98	1.28	1.11	0.97	1.26
	(Ref = unemployed)							
Health behaviors	Current smoking (Ref = Yes)		**1.27**	**1.06**	**1.53**	**1.28**	**1.07**	**1.54**
	Drinking alcohol (Ref = Yes)		0.90	0.79	1.04	0.90	0.78	1.03
	Aerobic physical activity (Ref = No)		**1.28**	**1.13**	**1.46**	**1.27**	**1.11**	**1.44**
	Ambulatory service use (Ref = No)		1.13	0.99	1.28	1.13	0.99	1.28
Current health status	Self-rated health		**1.86**	**1.04**	**3.32**	**1.90**	**1.06**	**3.40**
	EQ-5D		**7.43**	**1.66**	**33.33**	**7.13**	**1.56**	**32.47**
Predisposing or co-morbidities	Cancer (Ref = No)	Yes	1.02	0.84	1.23	1.00	0.82	1.21
	Stroke (Ref = No)	Yes	0.92	0.61	1.41	0.97	0.64	1.47
	Obesity (Ref = No)	Yes	**0.85**	**0.74**	**0.97**	**0.85**	**0.74**	**0.98**
	No. of chronic diseases		1.01	0.95	1.07			
	Hypertension (Ref = No)	Yes	–	–	–	**3.14**	**1.36**	**7.21**
	Dyslipidemia (Ref = No)	Yes	–	–	–	1.15	0.96	1.37
	Arthritis (Ref = No)	Yes	–	–	–	1.02	0.84	1.23
	Osteoporosis (Ref = No)	Yes	–	–	–	**1.47**	**1.11**	**1.95**
	Asthma (Ref = No)	Yes	–	–	–	**0.44**	**0.27**	**0.73**
	Thyroid disorder (Ref = No)	Yes	–	–	–	1.15	0.79	1.69

Regarding health behaviors, non-smokers were 1.27 times more likely take dietary supplements than smokers (OR = 1.27; 95% CI, 1.06–1.53), and those who practice aerobic physical activities were 1.28 times more likely to take dietary supplements (OR = 1.28; 95% CI, 1.13–1.46).

Subjective health status, including self-rated health and HRQoL, increased the likelihood of dietary supplement intake by 1.86 (OR = 1.86; 95% CI, 1.04–3.32) and 7.77 times (OR = 7.77; 95% CI, 1.66–33.40), respectively.

Predisposing factors or comorbidities such as the number of chronic diseases, cancer, and stroke were not significantly associated with dietary supplement intake, except obesity. Those with obesity tended to adopt fewer dietary supplements (OR = 0.85; 95% CI, 0.74–0.97).

Model II considers specific chronic diseases that affect the health of the study population. The main results were consistent to those in model I. Participants who were female (OR = 1.81; 95% CI, 1.55–2.11), older (OR = 1.06; 95% CI, 1.04–1.07), highly educated, married (OR = 5.33; 95% CI, 2.84–10.00), and higher-income earners were found to have higher likelihood of taking dietary supplements. Family size remained associated with a lower likelihood of the intake (OR = 0.88; 95% CI, 0.83–0.94).

Similar to model I, the likelihood of persistently taking dietary supplements was higher for those who took care of their health by refraining from smoking (OR = 1.28; 95% CI, 1.07–1.54) and practicing physical activities (OR = 1.27; 95% CI, 1.11–1.44). Other variables such as self-rated health status and EQ-5D index scores significantly increased the likelihood of dietary supplement intake by 1.90 (95% CI 1.06–3.40) and 7.13 (95% CI 1.56–32.47), respectively. Those with obesity tended to less consume dietary supplements (OR = 0.85; 95% CI, 0.74–0.98). Among the specified chronic diseases, hypertension, osteoporosis, and asthma were significantly associated. Hypertension and osteoporosis increased the likelihood of the dietary supplements intake by 3.14 (OR = 3.14; 95% CI, 1.36–7.21) and 1.47 times (OR = 1.47; 95% CI, 1.11–1.95), respectively, whereas those with asthma were 0.44 times (OR = 0.44; 95% CI, 0.27–0.73) less likely to take dietary supplements.

## Discussion

This study aimed to investigate the factors contributing to the intake of dietary supplements in Korea using the nationwide representative KNHANES data. To this end, a case–control design was established by comparing a group of adults that consistently consumed dietary supplements for > = 1 year with a group that did not. A holistic approach that encompassed various aspects, such as the demographic and socioeconomic factors of the study participants, health behavior, self-rated health, and morbidity, was attempted. Considering these multidimensional factors, we sought a comprehensive understanding of those associated with dietary supplement consumption in Korea.

Our findings confirm that sociodemographic factors significantly affect the intake of dietary supplements. Women were more likely to adopt dietary supplement than men, which is consistent with previous studies (14–19); however, Choi et al. found opposite results in Korean adults (22). In addition, our study found that older, married, well-educated, and high-income earners were more likely to consume dietary supplements, which is consistent with previous studies (14–19). This is likely due to the fact that these socioeconomic factors play a role in increasing interest in health, seeking information, and purchasing dietary supplements for their well-being.

Additionally, healthy behaviors and health status were found to play a significant role in supplement consumption. People in good health were more likely to pursue health and well-being by engaging in healthy behaviors, such as exercising and not smoking, and they tended to rate their health as relatively good. Smoking was significantly associated with lower subjective health status (30–35). Thus, individuals with high subjective health levels were more likely to make an effort to manage their health. Functional foods or dietary supplements are consumed in the same context. As shown in our findings, those who engaged in healthy behaviors and rated their health as good tended to consume more dietary supplements, which is consistent with the previous study (18). However, this is still controversial; some studies have revealed that health-oriented people were more likely to adopt functional food as a complementary health practice to lead healthy lives (16, 19), whereas smokers or those with poor subjective health were more likely to take functional foods or dietary supplements (12, 17, 18). However, our findings confirmed that the consumption of dietary supplements could be identified as part of health promotion, an individual’s effort to improve health, which is consistent with previous studies (16, 18–20, 36).

Regarding the morbidity of the study participants, we performed logistic regression using two models. As a result, the intake of dietary supplements was not significantly associated with health vulnerability. Presumably, we assumed that those who had chronic diseases or previously experienced serious diseases might be more inclined to consume dietary supplements. However, our findings did not support this assumption. In model I, which considered the number of chronic diseases, only obesity was significant, confirming that the more obese people were, the less they consumed dietary supplements (OR = 0.85; 95% CI, 0.74–0.97). This could be interpreted that people who are more health conscious are more likely to be concerned about improving their health, including weight management, and that these people consume more dietary supplements. This is consistent with Bailey et al. study (18). In model II, which included significant chronic diseases in the univariate regression analysis, obesity (OR = 0.85; 95% CI, 0.74–0.98), hypertension (OR = 3.14; 95% CI, 1.36–7.21), osteoporosis (OR = 1.47; 95% CI, 1.11–1.95), and asthma (OR = 0.44; 95% CI, 0.27–0.73) showed a significant association with consumption of dietary supplements. People with hypertension, and osteoporosis consumed more dietary supplements but those with obesity and asthma did not. This is partly consistent with other studies: Mahdavi-Roshan et al. found that people with diabetes, hypertension, cardiovascular disease, and obesity adopted more dietary supplements (14), and Choi et al. revealed that people with disabilities or chronic diseases and those who were hospitalized or who consulted doctors consumed more dietary supplements (22).

This study has several limitations. First, as stated earlier, we had difficulty in choosing the term “dietary supplement,” which is used literally in the KNHANES and is not different from, but includes functional foods in Korea. People’s perceptions of dietary supplements and functional foods are important since the definition of these terms differs from country to country. While there are arguments for a clear distinction between the two terms, they are perceived identically, at least by Koreans. Therefore, this study treated them as the same as they are perceived by the ordinary Koreans, which may not be acceptable in other contexts. However, for a more accurate investigation, it is necessary to provide a clear definition of dietary supplements in the future or to revise them to the official regulatory term “functional food.” Second, cross-sectional data were used. To identify the factors influencing the intake of dietary supplements, cross-sectional data should be interpreted with caution. Therefore, further studies using longitudinal data are required. Third, there are no existing systematic studies on the association between morbidity/prevalence and dietary supplement intake; therefore, more detailed studies are needed in the future. Finally, some of the results of this study differ from those of previous studies. In particular, previous studies on Koreans showed that chronic diseases or disabilities, medical visits, or hospitalizations had a significant effect on dietary supplement intake; however, this study did not show consistent results. However, given that our research was based on nationwide representative data, which usually allows for the generalization of results, our study yielded more reliable and valid results.

Despite these limitations, it is worth noting that our study was the first to investigate factors contributing to dietary supplement consumption in Korea using nationally representative data. In contrast to existing research, we investigated the factors that influence dietary supplement intake through a systematic approach by considering not only sociodemographics and health behaviors but also subjective health status, HRQoL, and comorbidities or predisposing diseases. In particular, the current prevalence or subjective health status, including HRQoL, which was not considered in previous studies, was explored in our study. Stroke, cancer, and myocardial infarction/angina, which are generally considered to have a tremendous impact on health, were not significant; however, hypertension, osteoporosis, obesity, and asthma were. Further studies are needed to identify the impact of the experience of disastrous diseases on health-promoting behaviors (i.e., dietary supplement consumption) using longitudinal data. Subjective health, HRQoL, and healthy behaviors were positively associated with dietary supplement consumption.

In conclusion, our study confirmed, as identified by previous studies (11, 16, 18–21, 36, 37), that the consumption of dietary supplements can be understood in the same context as individual health promotion activities for well-being or healthy living among Koreans. Given that the purpose of dietary supplements is to provide a positive impact on health and well-being beyond their nutritive content, dietary supplement policy targeting Koreans should focus on ensuring the safety and quality of dietary supplements as well as providing accurate information to consumers in the area of health promotion rather than therapeutic effects.

## Data availability statement

Publicly available datasets were analyzed in this study. This data can be found at: https://knhanes.kdca.go.kr/knhanes/main.do.

## Author contributions

H-YK conceived the study design, analyzed the data, and drafted and finalized the manuscript.

## Funding

This work was supported by the Ministry of Food and Drug Safety (MFDS) under Grant 23192-058.

## Conflict of interest

The author declares that the research was conducted in the absence of any commercial or financial relationships that could be construed as a potential conflict of interest.

## Publisher’s note

All claims expressed in this article are solely those of the authors and do not necessarily represent those of their affiliated organizations, or those of the publisher, the editors and the reviewers. Any product that may be evaluated in this article, or claim that may be made by its manufacturer, is not guaranteed or endorsed by the publisher.

## Footnotes

^1^ https://knhanes.kdca.go.kr

## References

[1] Wróbel K Milewska AJ Marczak M Kozłowski R. The impact of the COVID-19 pandemic on the composition of dietary supplements and functional foods notified in Poland. Int J Environ Res Public Health. (2021) 18:11751. doi: 10.3390/ijerph182211751, PMID: 34831505 PMC8622621

[2] Statista. Size of the worldwide market for dietary supplements from 2018 to 2028, vol. 21 Published by Nils-Gerrit Wunsch (2021). Available at: www.statistica.com.

[3] OECD. Organization for Economic co-operation and Development. OECD Data. (2022). Available at: https://data.oecd.org.

[4] KOSIS. Korean statistical information service. Available at: www.kosis.kr. (Accessed May 20, 2023).

[5] MFDS. Ministry of food and drug safety Statistics of functional food industry (2020).

[6] Phillips MM Rimmer CA. Functional foods and dietary supplements. Anal Bioanal Chem. (2013) 405:4323–4. doi: 10.1007/s00216-013-6846-9 23494275

[7] FDA. Food and drug administration. Available at: https://www.fda.gov/drugs/frequently-asked-questions-popular-topics/dietary-supplements-questions-and-answers.

[8] Binns C Lee M Lee A. Problems and prospects: public health regulation of dietary supplements. Annu Rev Public Health. (2018) 39:403–20. doi: 10.1146/annurev-publhealth-040617-013638, PMID: 29272167

[9] Korean Functional food Act. Available at: www.law.go.kr.

[10] KDCA Korea Disease Control and Prevention Agency. Guidelines for the 9th Korean National Health and nutrition examination survey (KNHANES) (2022–2024). Seoul: Ministry of Health and Welfare & Korea Centers for Disease Control and Prevention. 2019.

[11] Ozen AE Pons A Tur JA. Worldwide consumption of functional foods: a systematic review. Nutr Rev. (2012) 70:472–81. doi: 10.1111/j.1753-4887.2012.00492.x, PMID: 22835140

[12] de Jong N Ocké MC Branderhorst HAC Friele R. Demographic and lifestyle characteristics of functional food consumers and dietary supplement users. Br J Nutr. (2003) 89:273–81. doi: 10.1079/bjn2002772, PMID: 12575912

[13] Herath D Cranfield J Henson S. Who consumes functional foods and nutraceuticals in Canada? Results of cluster analysis of the 2006 survey of Canadians’ demand for food products supporting health and wellness. Appetite. (2008) 51:256–65. doi: 10.1016/j.appet.2008.02.018, PMID: 18417254

[14] Mahdavi-Roshan M Rezazadeh A Joukar F Khorshidi Y Naghipour M Mansour-Ghanaei F. Dietary supplements consumption and its association with socioeconomic factors, obesity and main non-communicable chronic diseases in the north of Iran: the PERSIAN Guilan cohort study (PGCS). BMC Nutr. (2021) 7:84. doi: 10.1186/s40795-021-00488-2 34906216 PMC8672625

[15] Kraus A Annunziata A Vecchio R. Sociodemographic factors differentiating the consumer and the motivations for functional food consumption. J Am Coll Nutr. (2017) 36:116–26. doi: 10.1080/07315724.2016.1228489, PMID: 28067592

[16] Niva M. Can we predict who adopts health-promoting foods? Users of functional foods in Finland. Scand J Food Nutr. (2006) 50:13–24. doi: 10.1080/11026480600655378

[17] Markovina J Čačić J Gajdoš Kljusurić J Kovačić D. Young consumers’ perception of functional foods in Croatia. Br Food J. (2011) 113:7–16. doi: 10.1108/00070701111097303

[18] Bailey R Gahche J Miller P Thomas P Dwyer J. Why US adults use dietary supplements. JAMA Intern Med. (2013) 173:355–61. doi: 10.1001/jamainternmed.2013.2299, PMID: 23381623

[19] Mullie P Guelinckx I Clarys P Degrave E Hulens M Vansant G. Cultural, socioeconomic and nutritional determinants of functional food consumption patterns. Eur J Clin Nutr. (2009) 63:1290–6. doi: 10.1038/ejcn.2009.89, PMID: 19707227

[20] Frewer L Scholderer J Lambert N. Consumer acceptance of functional foods: issues for the future. Br Food J. (2003) 105:714–31. doi: 10.1108/00070700310506263

[21] Dickinson A Blatman J El-Dash N Franco JC. Consumer usage and reasons for using dietary supplements: report of a series of surveys. J Am Coll Nutr. (2014) 33:176–82. doi: 10.1080/07315724.2013.875423, PMID: 24724775

[22] Choi JH You CH Kwon YD. Effects of health-related factors on the use of health functional foods. Korean J Health Serv Manag. (2011) 5:27–39. doi: 10.12811/kshsm.2011.5.4.027

[23] Park KC Choi YH Kim WR Choi YJ Yoon KS. Intake status and recognition of health functional foods by pre-and post-menopausal women in Seoul and Gyeonggi province. J Korean Soc Food Sci Nutr. (2014) 43:1112–21. doi: 10.3746/jkfn.2014.43.7.1112

[24] Korea Disease Control and Prevention Agency (KDCA). The seventh Korea national health and nutrition examination survey (KNHANES VII) 2016–2018. Seoul: Ministry of Health and Welfare & Korea Centers for Disease Control and Prevention (2007).

[25] Korea Disease Control and Prevention Agency (KDCA). The 8th Korea national health and nutrition examination survey (KNHANES VIII) 2020–2022. Seoul: Ministry of Health and Welfare & Korea Centers for Disease Control and Prevention (2020).

[26] Korea Disease Control and Prevention Agency (KDCA), Guidelines for the use of raw data from the 8th KNHANES (2019–2021). Seoul: Ministry of Health and Welfare & Korea Centers for Disease Control and Prevention. 2019.

[27] Nam HS. (2010). South Korean time trade-off values for EQ-5D health states. Available at: http://www.cdc.go.kr/CDC/info/CdcKrInfo0301.jsp?menuIds=HOME001-MNU1132-MNU1138-MNU0037-MNU1380&cid=12449. 10.1111/j.1524-4733.2009.00579.x 19659703

[28] Lee YK Nam HS Chuang LH Kim KY Yang HK Kwon IS. South Korean time trade-off values for EQ-5D health states: modeling with observed values for 101 health states. Value Health. (2009) 12:1187–93. doi: 10.1111/j.1524-4733.2009.00579.x, PMID: 19659703

[29] Park YG. Comments on statistical issues in march 2014. Korean J Fam Med. (2014) 35:107–8. doi: 10.4082/kjfm.2014.35.2.107, PMID: 24724006 PMC3978184

[30] Li C Khan MM. Smoking and self-rated health status of older men in China. Aging Health Res. (2022) 2:100050–8. doi: 10.1016/j.ahr.2021.100050

[31] Abuladze L Kunder N Lang K Vaask S. Associations between self-rated health and health behaviour among older adults in Estonia: a cross-sectional analysis. BMJ Open. (2017) 7:e013257. doi: 10.1136/bmjopen-2016-013257, PMID: 28601816 PMC5734211

[32] Manderbacka K Lundberg O Martikainen P. Do risk factors and health behaviours contribute to self-ratings of health? Soc Sci Med. (1999) 48:1713–20. doi: 10.1016/S0277-9536(99)00068-4, PMID: 10405010

[33] Lim WY Ma S Heng D Bhalla V Chew SK. Gender, ethnicity, health behaviour & self-rated health in Singapore. BMC Public Health. (2007) 7:184. doi: 10.1186/1471-2458-7-184, PMID: 17655774 PMC1976324

[34] Prus SG. Comparing social determinants of self-rated health across the United States and Canada. Soc Sci Med. (2011) 73:50–9. doi: 10.1016/j.socscimed.2011.04.010, PMID: 21664020

[35] Svedberg P Bardage C Sandin S Pedersen NL. A prospective study of health, life-style and psychosocial predictors of self-rated health. Eur J Epidemiol. (2006) 21:767–76. doi: 10.1007/s10654-006-9064-3, PMID: 17106761

[36] Goetzke B Nitzko S Spiller A. Consumption of organic and functional food. A matter of well-being and health? Appetite. (2014) 77:96–105. doi: 10.1016/j.appet.2014.02.012, PMID: 24630940

[37] Schroeder D. Public health, ethics, and functional foods. J Agric Environ Ethics. (2007) 20:247–59. doi: 10.1007/s10806-007-9033-1

